# Anesthetic Properties of Lycoperdon Proteus

**Published:** 1853-10

**Authors:** 


*Anesthetic Properties of Lycoperdon Proteus (Common Puff Ball).*—The number of the *Medical Times and Ga-*

*zette* for June 11 contains an abstract of a paper read before the Medical Society of London, on the anesthetic properties of the lycoperdon proteus. The author's attention had been directed to the fact, that the smoke of the common puff-ball was used in the country for stupifying bees, and the idea struck him that it would be worth while to ascertain if the same agent would produce narcotism in the higher classes of animals. Several weeks since he commenced a series of experiments with the fumes of the fungus, and had continued them to the present time. He found it possible to produce the most perfect anesthesia with the fumes. His experiments were made on dogs, cats, and rabbits, and had been witnessed by Drs. Willis, Crisp, Cormack, Snow, and several others. He had administered the narcotic fumes in the impure state, and in a clarified state obtained by passing them through a solution of caustic potass. When an animal was exposed to a large quantity of the narcotic vapor, the narcotism came on very speedily, and the insensibility was most decided, but recovery soon took place. Dr. Willis and Mr. Richardson had removed a large tumor from the abdomen of a dog that had been placed under the influence of the narcotic. No sign of pain was shown during the operation, and the animal did well afterwards The fumes were obtained by burning the fungus. When a moderate quantity was inhaled slowly, the narcotism came on and passed off slowly, the animal exhibiting all the symptoms of intoxication, with convulsions, and sometimes vomiting. Several animals had been intentionally destroyed by the narcotic. It destroyed life slowly ; a dog would often inhale the fumes for twenty minutes or half an hour after being completely narcotized, previous to expiring. The heart's beat in all cases survived the respirations. The lungs after death were pale ; there was no sign of congestion in any organ ; the blood retained its red color, but did not coagulate quickly; cadaveric rigidity set in in two or three hours. During recovery from a protracted narcotism, an animal would sometimes be quite conscious, but insensible to pain. Mr. Richardson had himself inhaled the clarified fumes of the fnngus ; they produced in him symptoms of intoxication and drowsiness, but he did not breathe them long enough to become completely narcotized. Mr. Richardson was able to afford but little information as to the nature of the narcotic agent contained in the fumes. Many of the fungi possessed narcotic properties, and had been supposed to possess an alkaloid resembling morphia; but the subject had never been thoroughly investigated. He should only say, concerning the narcotic principle contained in the puff-ball —

1st. That it was of a most volatile nature.

2d. That it was not absorbed by alcohol, water, or strong alkaline solution.

3d. That if the fungus was burned in oxygen gas, the narcotic principles still remained in the fumes, and produced its effect, if free oxygen was breathed with it.

The fungus had been given internally to two animals without effect. In Italy, it was fried and eaten as food. In conclusion, Mr. Richardson said, that he had been anxious only to show that a volatile narcotic principle, capable of causing anesthesia by inhalation, did exist in one of the fnngi; it remained to be seen whether other fungi possessed a similar principle, and whether from a fungus an anesthetic could be obtained that might be useful in practice, with as little trouble to the operator and with less danger to the patient than ether or chloroform.

Dr. Snow corroborated Mr. Richardson's observations, having witnessed several of his experiments. There could be no doubt that the fungus did possess a very volatile narcotic principle, capable of causing insensibility to pain. As yet, however, the narcotic was not so practicable as chloroform. The subject deserved and required farther search.—*Am. Jour. Med. Sci.*

